# Effects of reducing, stabilizing, and antibiotic agents on “*Candidatus* Kuenenia stuttgartiensis”

**DOI:** 10.1007/s00253-023-12375-w

**Published:** 2023-02-08

**Authors:** Emea Okorafor Ude, Jucelaine Haas, Mohammed Kaysar Kaiyoum, Chang Ding, Lorenz Adrian

**Affiliations:** 1 Department of Environmental Biotechnology, Helmholtz Centre for Environmental Research - UFZ, Permoserstraße 15, 04318 Leipzig, Germany; 2 Biological Control Laboratory, Federal University of Technology - Parana - UTFPR Campus Dois Vizinhos, Estrada Para Boa Esperança Km 4, Dois Vizinhos, 85660-000 Brazil; 3 Chair of Geobiotechnology, Technische Universität Berlin, Ackerstraße 76, 13355 Berlin, Germany

**Keywords:** Anammox bacteria, Antibiotics, Online monitoring, Planktonic cell, Reducing agents, Sulfite

## Abstract

**Abstract:**

Anaerobic ammon ium oxidizing (anammox) bacteria oxidize ammonium and reduce nitrite, producing N_2_, and could play a major role in energy-optimized wastewater treatment. However, sensitivity to various environmental conditions and slow growth currently hinder their wide application. Here, we attempted to determine online the effect of environmental stresses on anammox bacteria by using an overnight batch activity test with whole cells, in which anammox activity was calculated by quantifying N_2_ production via headspace-pressure monitoring. A planktonic mixed culture dominated by “*Candidatus* Kuenenia stuttgartiensis” strain CSTR1 was cultivated in a 30-L semi-continuous stirring tank reactor. In overnight resting-cell anammox activity tests, oxygen caused strong inhibition of anammox activity, which was reversed by sodium sulfite (30 µM). The tested antibiotics sulfamethoxazole, kanamycin, and ciprofloxacin elicited their effect on a dose-dependent manner; however, strain CSTR1 was highly resistant to sulfamethoxazole. Anammox activity was improved by activated carbon and Fe_2_O_3_. Protein expression analysis from resting cells after anammox activity stimulation revealed that NapC/NirT family cytochrome c (KsCSTR_12840), hydrazine synthase, hydrazine dehydrogenase, hydroxylamine oxidase, and nitrate:nitrite oxidoreductase were upregulated, while a putative hydroxylamine oxidoreductase HAO (KsCSTR_49490) was downregulated. These findings contribute to the growing knowledge on anammox bacteria physiology, eventually leading to the control of anammox bacteria growth and activity in real-world application.

**Key Points:**

• *Sulfite additions can reverse oxygen inhibition of the anammox process*

• *Anammox activity was improved by activated carbon and ferric oxide*

• *Sulfamethoxazole marginally affected anammox activity*

**Graphical abstract:**

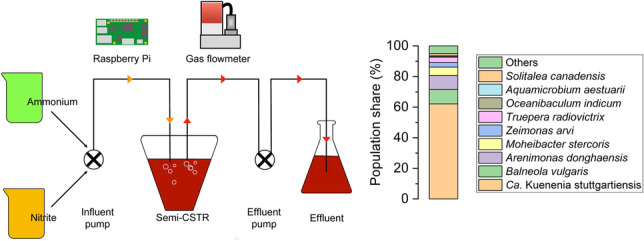

**Supplementary Information:**

The online version contains supplementary material available at 10.1007/s00253-023-12375-w.

## Introduction

Anaerobic ammonium oxidizing (anammox) bacteria depend on specific environmental conditions for their metabolic activities and growth. They use ammonium as electron donor and nitrite as electron acceptor to produce dinitrogen gas in anaerobic respiration (van de Graaf et al. 1995; van Niftrik and Jetten 2012). The energy from this reaction (∆G°' =  − 357 kJ mol^−1^) is fixed through a series of biochemical reactions and used for autotrophic growth (de Almeida et al. 2016; Kartal et al. 2013). Owing to the low energy available from the reaction, the molar growth yield (cells per mole of substrate) is low compared to that of other biological processes such as aerobic respiration; therefore, the turnover of substrates per cell cycle is high. Anammox bacteria remove fixed nitrogen compounds without energy-intensive aeration or amendment of external carbon sources (Sonthiphand et al. 2014), and it has often been proposed to integrate an anammox stage in wastewater treatment plants for nitrogen removal (Hauck et al. 2016). Whereas many wastewater treatment plants are using an anammox stage for the treatment of ammonium-rich side streams, current developments aim to stably establish the anammox process also in the mainstream of wastewater treatment plants (Trinh et al. 2021).

“*Candidatus* Kuenenia stuttgartiensis” is the best characterized anammox bacterium among the described genera, and its metabolism has been extensively studied (van Niftrik and Jetten 2012). In the energy metabolism of *Ca.* K. stuttgartiensis, nitrite is first reduced (one electron) to the radical nitric oxide (NO), which combines with ammonium and further three electrons to form hydrazine in a second reaction, catalyzed by hydrazine synthase (HZS). Hydrazine is oxidized (four electrons) to N_2_ by hydrazine dehydrogenase (HDH), and the electrons are pumped back to the cytoplasm to recover the electrons lost during the previous reduction steps. The anammox metabolism depends highly on heme proteins, e.g., the multi-heme enzymes HZS and HDH, quinol:cytochrome c oxidoreductase (complex III, bc1) (Strous et al. 2006), and cytochrome c as an electron mediator. The presence of such proteins in high amounts explains the reddish color of enriched anammox cultures (Kartal and Keltjens 2016).

Anammox bacteria have been broadly regarded as slow growers, limiting their application (e.g., in wastewater technology). Growth rates of 0.06–0.08 d^−1^ and doubling times of 8–11 days have been described for *Ca.* K. stuttgartiensis (van der Star et al. 2008). However, in recent years, a specific growth rate as high as 0.33 d^−1^ has been achieved when anammox cells were grown planktonically, either in a membrane bioreactor (Lotti et al. 2015) or in a semi-continuous stirred tank reactor (semi-CSTR) (Ding et al. 2018). In the semi-CSTR, biomass retention was waivered in favor of culture homogeneity, cultivation reproducibility, and higher cell growth rate. It is important to have in mind that this approach was not developed for application in wastewater treatment but for physiological characterization of the cells. The semi-CSTR allowed not only the production of planktonic cells under strictly anoxic conditions but also real-time monitoring of the effect of abiotic parameters on anammox activity. The reactor thus provides the perfect tool for detailed, in-depth physiological studies on anammox bacteria under diverse conditions and stresses.

Despite the widespread interest in anammox bacteria, the fast and reliable start-up of the anammox system is still the bottleneck for its broad practical application in wastewater treatment. One limiting factor could be that dedicated enrichment and cultivation techniques are required (Kartal et al. 2012). Laboratory studies have cultivated anammox bacteria in small reactor volumes (Manonmani and Joseph 2018; Miao et al. 2020; Reino et al. 2018; Yi et al. 2011; Zhang et al. 2019a), but upscaling the cultivation of anammox bacteria to a large volume often resulted in intricacies that led to reactor failures (Nielsen et al. 2005; Wett 2006).

Importantly, in wastewater treatment plants, anammox bacteria are confronted with a wide range of potential inhibitors, e.g., oxygen (Strous et al. 1997; Zhang and Okabe 2020) and antibiotics (Madeira and de Araújo 2021), that can impact their growth and activity. Protection from or reversion of the oxygen effect is key for implementation of the anammox process, but it has not been extensively studied. Reducing agents at appropriate concentrations can eliminate molecular oxygen, thereby providing favorable anoxic conditions for anammox bacteria. Antibiotics are a second important environmental factor because many industrial wastewaters rich in ammonium (such as pharmaceutical, municipal, and animal farm wastewater, and black water) also contain antibiotics (Liang et al. 2015). Antibiotics can affect microbial activities in wastewater treatment (Zhang et al. 2019b), which has been shown previously (Lu et al. 2021; Madeira and de Araújo 2021), albeit all studies have been performed with sludge granules that intrinsically protect the enclosed bacteria. In contrast to inhibitors, many effectors such as graphene, ferrous iron, or manganese can enhance anammox activity (Bi et al. 2014; Ganesan and Vadivelu 2019; Wang et al. 2013; Wu et al. 2020; Yin et al. 2016), but their link with the inherent abilities (genes and proteins) of anammox bacteria are lacking in the literature. It is important to know the molecular mechanisms through which these effectors influence gene expression to rationally design optimized conditions for anammox bacteria growth in real applications.

Here, we aimed to upscale planktonic cultivation of the anammox bacterium *Ca.* K. stuttgartiensis strain CSTR1 and to physiologically characterize the cells from this reactor. As the growth of strain CSTR1 is tightly linked to the anammox activity, we hypothesized that we could determine the effect of environmental parameters in an overnight batch activity test with whole cells, quantifying N_2_ production by headspace-pressure monitoring. By adopting this setup, we were able to use the reactor effluent in 12 individual small batch bottles and monitor the activity online directly with 12 individual low-cost pressure sensors via an analog/digital converter and a microprocessor. The system delivered data overnight and allowed reliable, simultaneous monitoring of parameters with negative and positive control bottles in triplicates. With this system, we observed the response of strain CSTR1 to oxygen, sulfite, cysteine, sodium thioglycolate, antibiotics, activated carbon, Fe_2_O_3_, MnCl_2_, and MnO_2_. Apart from monitoring headspace pressure, we evaluated cell growth, ammonium and nitrite consumption, nitrate and gas production, as well as responses in protein expression.

## Materials and methods

### Production of an inoculum of strain CSTR1

We started our work with a mixed bacterial culture containing predominantly *Ca.* K. stuttgartiensis strain CSTR1, grown under anoxic conditions as planktonic cells in a 1-L semi-CSTR as previously described (Ding and Adrian 2020; Ding et al. 2018). The sludge-free effluent of this 1-L reactor, containing planktonic cells, was directly captured in a glass bottle located in a small fridge beside the reactor to preserve the viability of the cells. This refrigerated effluent later served as a seed culture for the upscaled 30-L reactor.

### Cultivation of strain CSTR1 in an upscaled 30-L reactor

A polyethylene reactor with a total volume of 35 L and a liquid volume of 30 L was established to obtain enough biomass for all subsequent experiments. The reactor components and operation in the 30-L reactor were similar to those previously described for a small laboratory-scale reactor (2.8 L volume) (Ding et al. 2018), with small modifications: The reactor had one port for medium amendment, sampling, and effluent discharge and one port for a pressure sensor; evolving gas in the headspace was released to the atmosphere via a flow meter (F-100D, Bronkhorst, Ruurlo, Netherlands); and the data of the flow meter was used for online quantification of anammox activity. The media feeding solutions were connected via two peristaltic pumps (ISM597D, Cole-Parmer, Wertheim, Germany) to semi-continuously feed the reactor. A third peristaltic pump was connected to one of the ports of the reactor to pump out the effluent. All operations were performed automatically and controlled via a Raspberry Pi microprocessor (Ding et al. 2018). Samples were taken through the effluent port.

To start the 30-L reactor, it was filled with 28 L of fully synthetic anoxically prepared start-up medium, containing 3 mM nitrite and 3 mM ammonium (Table S1). The reactor headspace was flushed with nitrogen until the oxygen concentration in the headspace was lower than 0.4%. Then, a 2 M aqueous solution of sodium sulfite was titrated to the medium to reduce the dissolved oxygen to 0.1 µM. To inoculate the reactor, 1.6 L of refrigerated effluent from the 1-L reactor (see “Production of an inoculum of strain CSTR1”) was added to obtain an initial cell density of 1.1 × 10^7^ mL^−1^.

The 30-L reactor was operated by feeding it with the 1:1 mixture of two synthetic mineral feeding media (Table S2a and S2b) resulting in a concentration of 60 mM ammonium and 60 mM nitrite in the inflow to the reactor. The nitrite-loading rate was increased gradually by increasing the influent flow rate of the feeding media as long as the reactor consumed the amended nitrite.

### Quantification of cell growth and heme proteins

Growth of the strain CSTR1 was monitored by epifluorescence microscopic direct cell counting and by determining the absorption of the effluent as a proxy for heme concentration and bacterial cell mass. For epifluorescence microscopy, cells were counted on low-melting agarose coated slides (Adrian et al. 2007) every 48 h from day 0 until the end of the experiment. An aliquot of 20 μL of liquid culture was stained with 1.3 μL of SYBR Green I solution (prepared by diluting 5 µL of the SYBR Green I stock solution (10,000 ×) with 495 µL sterile TE buffer) (Invitrogen Corporation, Eugene, OR, USA) and incubated at room temperature (21 ± 3 °C) for 10 min. After incubation, 18 μL of the stained culture was pipetted on a glass slide and covered with a 20 mm × 20 mm cover glass. The cell number was averaged in at least 15 different visual fields using an epifluorescence microscope (Optiphot 2, Nikon, Minato City, Japan). Pictures were taken using a Nikon DS-Vi1 digital camera. Each image covered an area of 154.59 μm in length and 115.92 μm in width and had a dimension of 1600 × 1200 pixels. Cell counting was achieved using the Particle Analysis function on ImageJ (https://imagej.nih.gov/ij/), which counted all particles with intensity > 50 and particle size 30–2000 pixels. Cells of *Ca*. K. stuttgartiensis have a characteristic C-shaped morphology that can be visually differentiated from that of other cells in the mixed community (Ding et al. 2018).

Heme absorbance was monitored using a spectrophotometer (UV-1800, Shimadzu 240 IVDD, Kyoto, Japan). Fresh samples (1.5 mL) were collected from the 30-L reactor every 48 h and transferred to quartz cuvettes. Absorption was measured between 300 and 800 nm, and heme peaks with maxima at 410 nm were monitored. The peak area (absorbance unit (au) × nm) was used for quantification. Heme was quantified at the end of the experiment by a high performance liquid chromatograph equipped with a diode array detector (HPLC–DAD; Ultimate 3000 HPLC, Dionex Softron GmbH, Germering, Germany) at the wavelength of 398 nm, using hemin as standard as described previously (Fyrestam and Östman 2017).

### Microbial community analysis

Comprehensive microbial community analysis was performed by 16S rRNA gene amplicon sequencing. For that, 1 mL of the liquid culture was centrifuged at 10,000 × *g* for 15 min at 4 °C and genomic DNA was extracted from the pellet using the DNA extraction kit DNeasy power soil kit (QIAGEN, Hilden, Germany) according to the manufacturer’s instructions. Extracted DNA samples were stored at − 20 °C and used for polymerase chain reaction (PCR) and Illumina sequencing of 16S rRNA genes. DNA metabarcoding analyses were carried out by AllGenetics & Biology SL (www.allgenetics.eu). For library preparation, a fragment of the bacterial 16S genomic region (approximately 500 bp) was amplified using the primers 338F_12 (5′ ACT CCT ACG GGA GGC WGC AG 3′), 338F_45 (5′ ACA CCT ACG GGT GGC WGC AG 3′), and 806R (5′ GGA CTA CHV GGG TWT CTA AT 3′) (Caporaso et al. 2012; Daims et al. 1999). A mixture of the forward primers (338F_12 and 338F_45) was used to cover most of the sequence variations in Bacteria including all fresh-water anammox bacteria (Ding et al. 2020). Library preparation and verification of the ligation was done as described previously (Vierna et al. 2017). Libraries were purified using the Mag-Bind RXNPure Plus magnetic beads (Omega Biotek, Norcross, GA, USA), following the instructions provided by the manufacturer. Then, libraries were pooled in equimolar amounts according to the quantification data provided by the Qubit dsDNA HS Assay (Thermo Fisher Scientific, Waltham, MA, USA). The pool was sequenced in a fraction of a MiSeq PE300 run (Illumina, San Diego, CA, USA). All sequence reads were processed using the NGS analysis pipeline of the SILVA rRNA gene database project (SILVAngs 1.3; (Quast et al. 2013)). The identification of organisms was performed using BLASTN at the NCBI webpage against the 16S ribosomal sequences (bacteria and archaea) database, which contains the 16S ribosomal RNA from curated type strain sequences from bacteria and archaea. In addition, the nucleotide collection (nt), in which strain CSTR1 is deposited, was used.

For fast monitoring the population share of strain CSTR1 in the reactor community, we applied denaturing gradient gel electrophoresis (DGGE). Samples for DNA extraction were taken from the reactor using a sterile plastic syringe, and cells were pelleted by centrifugation (twice at 10,000 × *g*, 16 °C, 30 min). Genomic DNA was extracted from the resulting pellet by using the NucleoSpin Tissue kit (Macherey–Nagel, Düren, Germany). PCR amplification of 16S rRNA genes was done with the GC-clamped forward primer 341FGC (5′-CGC CCG CCG CGC GCG GCG GGC GGG GCG GGG GCA CGG GGGG CCT ACG GGA GGC WGC AG- 3′) and the reverse primer 518R12 (5′-WTT ACC GCG GCT GCT GG-3′) (Ding et al. 2020). PCR products were checked on 1% agarose gels prior to DGGE analysis. The DGGE gel was prepared with 10% polyacrylamide and a gradient of 30–70% denaturing agents, where 100% corresponds to 7 M urea and 32% v/v formamide. A subsample of 20 µl of the amplified PCR products was loaded onto the polyacrylamide gel, and electrophoresis was run in 1 × TAE buffer at 100 V and 60 °C for 16 h in a DCode Universal Mutation Detection System (Bio-Rad, Hercules, CA, USA). The gel was SYBR-Gold stained, bands were cut, and DNA was eluted by overnight incubation in 10 mM Tris·HCl at pH 8.5. The eluted DNA was again PCR amplified with the primers 341FGC and 518R12. Finally, the PCR products were purified and sent for Sanger sequencing (Eurofins Genomics Europe, Ebersberg, Germany). The sequencing results were used for taxonomic identification by matching them against the NCBI nt database using nucleotide BLAST (http://www.ncbi.nlm.nih.gov/BLAST/).

### Resting-cell activity test

**Fig. 1:**
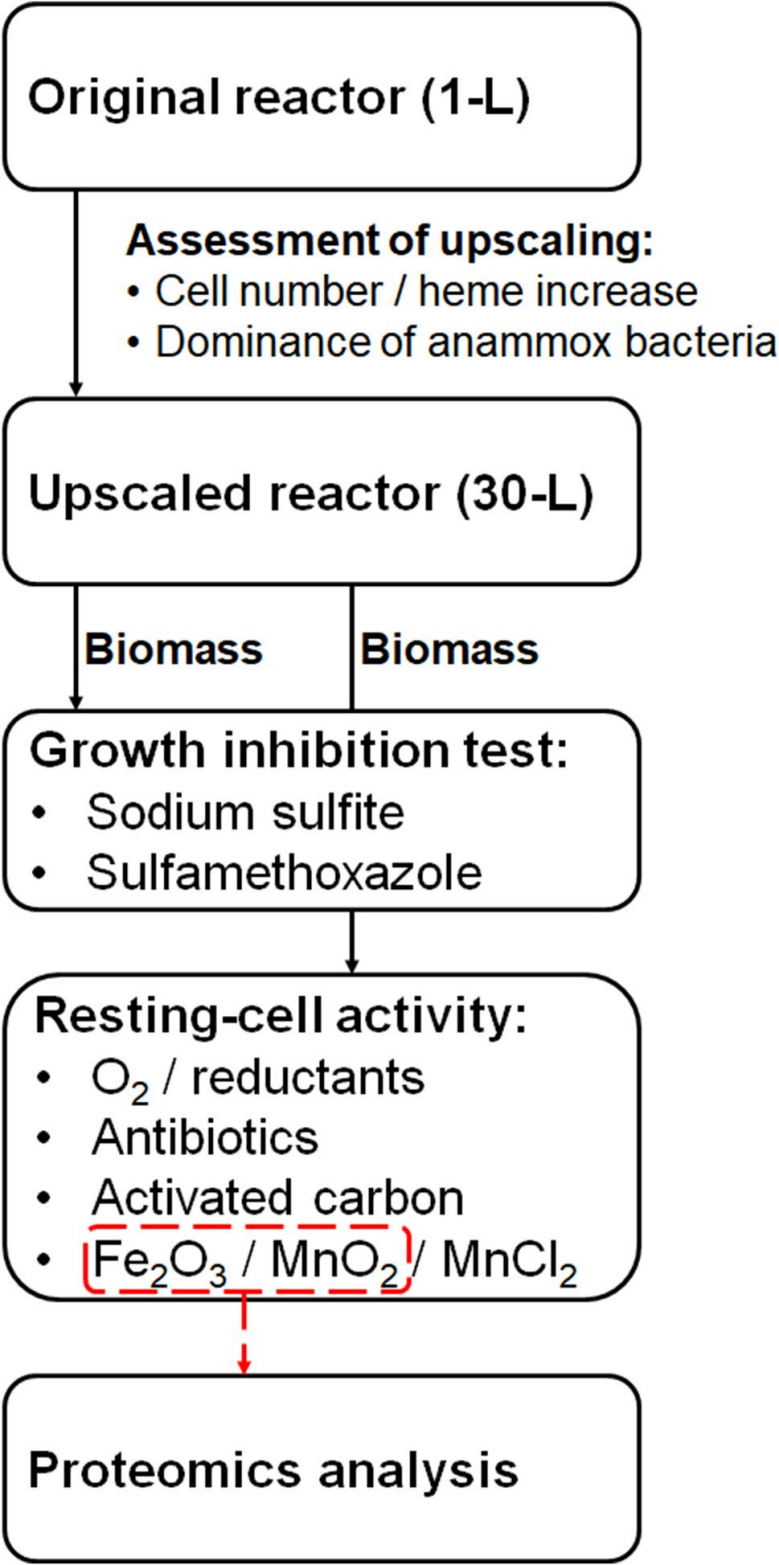
Experimental flow chart

Short-time exposure tests that were not dependent on cell growth were performed to investigate direct inhibition of enzymatic activity of whole cells. For such tests, the reactor effluent was incubated with the following compounds (for details of the experimental set-ups, see Table S3 and Fig. 1): O_2_ (0.3 mL air per mL headspace volume), cysteine (0–500 µM), sodium sulfite (0–100 µM), sodium thioglycolate (0–200 µM), sulfamethoxazole (0–2.5 mM), kanamycin (0–5 mM), ciprofloxacin (0–5 mM), activated carbon (purified, powdered charcoal, Chemviron Carbon, 0–1000 mg L^−1^), ferric oxide (Fe_2_O_3_, 0–12.6 mM), manganese (II) chloride (MnCl_2_∙4H_2_O, 0–0.5 mM), or manganese (IV) oxide (MnO_2_, 0–100 mg L^−1^). The two different Mn species were tested to analyze the effect of the two different redox states of Mn. Consumption of ammonium and nitrite and production of gas in headspace (as a proxy for N_2_) were monitored.

For each experiment, serum bottles (60 mL) were transferred into an anaerobic glovebox where they were filled with 50-mL sludge-free effluent from the 30-L reactor containing planktonic cells. Different concentrations of the tested effectors were then added to the bottles within the anaerobic glovebox (Table S3). The serum bottles were finally closed with butyl rubber septa and aluminum crimp caps. All experiments were performed in triplicate. For oxygen sensitivity/recovery experiments, 3 mL of air was injected into the bottles using a plastic syringe. The negative control bottles were amended with air only (no reducing agents). The positive control bottles were amended with 3 mL of nitrogen gas instead of air.

To determine gas production, each bottle was connected to a MPX5100DP pressure sensor (NXP Semiconductors, Eindhoven, Netherlands) via a 0.5-mm i.d. Luer Lock needle. All the sensors were electrically connected via a LabJack T7Pro analog/digital converter to a microprocessor (Raspberry Pi model 3B; (Ding et al. 2018)) for data reading and storage. Pressure sensors were read at 3-s intervals over the whole period of the experiment. The experiments ran continuously overnight. To stop the reaction, 3 mL of air were injected into each bottle. The pressure increased due to the injection of the 3 mL of air was also used to calibrate the pressure-sensor readings. The rate kinetics were calculated as described previously (Peng et al. 2017).

### Growth inhibition and recovery tests

Growth inhibition was evaluated in long-time exposure tests using 60-mL serum bottles. Purely synthetic medium containing sodium bicarbonate (pH buffer), 3 mM sodium nitrite, and 5.6 mM ammonium chloride were prepared as described previously in 45-mL aliquots in 60-mL serum bottles (Ding et al. 2018). The effector sodium sulfite or sulfamethoxazole was added in different concentrations as an aqueous solution or as a powder, respectively, before inoculating the cultures. Afterwards, active culture (5% (Table S4a) or 0.5% (Table S4b), v/v; final cell density of 9 × 10^6^ and 9 × 10^5^ cells mL^−1^, respectively) from the 30-L reactor was transferred into the 60-mL serum bottles. The positive control treatment contained bacteria but no effectors, and in the negative controls, bacteria were omitted. The no-bacterium controls served to rule out possible abiotic reactions of the effectors with ammonium or nitrite. Cultures were incubated without shaking at 30 °C for up to 4 weeks. The parameters evaluated were ammonium and nitrite consumption as well as nitrate production. All experiments were conducted in triplicate.

### Analytical techniques

Ammonium concentrations were determined with a modified salicylate method as described previously (Ding et al. 2018). Nitrate and nitrite concentrations were determined on a DX-120 ion chromatograph (Dionex, Sunnyvale, CA, USA) with an IonPac AS4A-SC column and an eluent containing 0.7 mM Na_2_CO_3_ and 0.7 mM NaHCO_3_ at a flow rate of 1 mL min^−1^.

### Differential protein expression analysis

To identify expressed proteins involved in respiratory energy conservation, a shotgun proteomics approach was applied. We tested the effects of Fe(III) and Mn(IV), which could potentially be used as respiratory electron acceptors, but not Mn(II), which cannot support anaerobic respiration. Cells exposed overnight in whole-cell activity test incubations to MnO_2_ or Fe_2_O_3_ were harvested by centrifugation at 10,000 × *g* for 30 min at 16 °C. The cells were resuspended in 50 mM ammonium bicarbonate buffer and subjected to another round of centrifugation under the same conditions. Washed cells were disrupted by three rounds of freezing (– 80 °C) and thawing (thermal shaker at 40 °C for 60 s and 750 rpm). Disrupted cells were then placed in an ultrasonic bath (A52103, Bandelin Digitec, Berlin, Germany) for 30 s. An anoxic aqueous solution of 700 mM dithiothreitol was added to a final concentration of 62.5 mM as a reducing agent to reduce disulfide bridges. The samples were incubated for 30 min at 37 °C and 300 rpm. A solution of 1 M 2-iodoacetamide was added to obtain a final concentration of 128 mM for cysteine acetamidylation, and the samples were incubated in the dark for 45 min at 300 rpm and 25 °C. After that, 5 µL of trypsin (0.1 µg µL^−1^) was added, and the samples were incubated overnight at 300 rpm and 37 °C. To stop the digestion, formic acid was added to a final concentration of 2%. Peptide samples were desalted using µC_18_ ZipTips (100 µL; Merck Millipore, Darmstadt, Germany). Desalted peptides were injected into a nanoLC-MS/MS system (Ultimate 3000 RS, Dionex) with an Orbitrap Fusion Tribrid mass spectrometer (MS; Thermo Fisher Scientific). Sample measurements were performed as described previously (Seidel et al. 2018).

### Data analyses

Raw data files from the LC–MS/MS analysis were processed using ProteomeDiscoverer version 2.4 (Thermo Fisher Scientific). All acquired data were processed to obtain means, standard deviations, and abundance percentages. Estimated abundances were obtained by using the Minora node implemented in ProteomeDiscoverer. The percentage values given in the text refer to the abundance of one particular protein divided by the sum of the abundances of all quantified proteins in a sample. Data were statistically analyzed using ANOVA, and means were compared with Tukey’s test at *p* < 0.05 significance threshold.

## Results

### Anammox bacteria were successfully enriched in the upscaled 30-L reactor

The overall experimental flow of our study is depicted in Fig. 1. As the basis for all experiments, we used a 1-L semi-CSTR with an enriched culture of planktonic cells of *Ca*. K. stuttgartiensis strain CSTR1. From the 1-L reactor, a 30-L semi-CSTR was established with a similar automatic process control as that of the 2.8-L reactor described previously (Ding et al. 2018). The initial medium contained 3 mM nitrite and 3 mM ammonium, and the reactor was incubated without flow until the pressure increase indicated anammox activity and nitrite concentration fell below 1 mM. Then the inflow of fresh medium containing 60 mM of nitrite and 60 mM of ammonium into the upscaled 30-L reactor was slowly started and gradually increased while maintaining the nitrite concentration below 1 mM. After 15 days of operation, the effluent turned reddish, and the reactor flow was gradually increased to 1 L d^−1^.

**Fig. 2:**
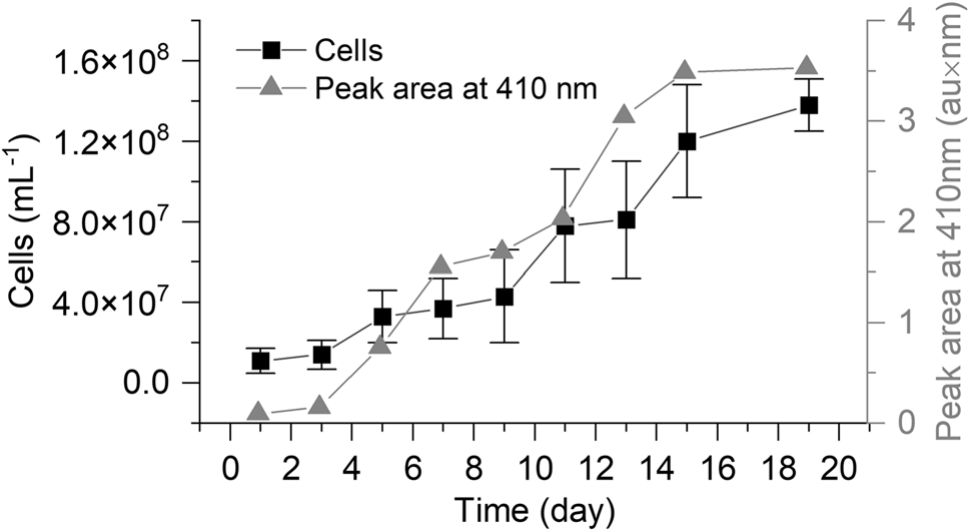
Cell count and heme concentration of strain CSTR1 in a 30-L semi-continuous-stirring tank reactor during enrichment. The black line (squares) represents cell density measured by epifluorescence microscopy (means ± SD; *n* = 3). The grey line (with triangles) represents the integrated peak area for the peak with a maximum at 410 nm monitored as a proxy for heme concentration

The enrichment process was monitored by measuring cell density via epifluorescence microscopy and heme-protein production by measuring the absorption peak at 410 nm (Fig. 2). Most cells found in the enriched effluent had a c-letter shape or ring shape typical for anammox bacteria. As indicated by periodical cell counting, the cell density of strain CSTR1 increased within the first 3 days, implying that the lag phase had been very short. Final cell numbers in the 30-L reactor reached 1.4 × 10^8^ cells mL^−1^. The area of the peak at 410 nm, indicative of heme within the cells, developed similarly and eventually reached 3.5 au × nm. There was a strong correlation (*R* = 0.97) between cell number (determined by epifluorescence microscopy) and heme absorption. At the end of the experiment, heme concentration was 0.16 ± 0.01 µM as quantified using HPLC–DAD. Simultaneously, DGGE analysis confirmed that the most abundant cells in the reactor were those of strain CSTR1 (Fig. S1). These results show that upscaling of cultivation to a 30-L scale was successfully achieved within a month maintaining the anammox-active community.

**Fig. 3:**
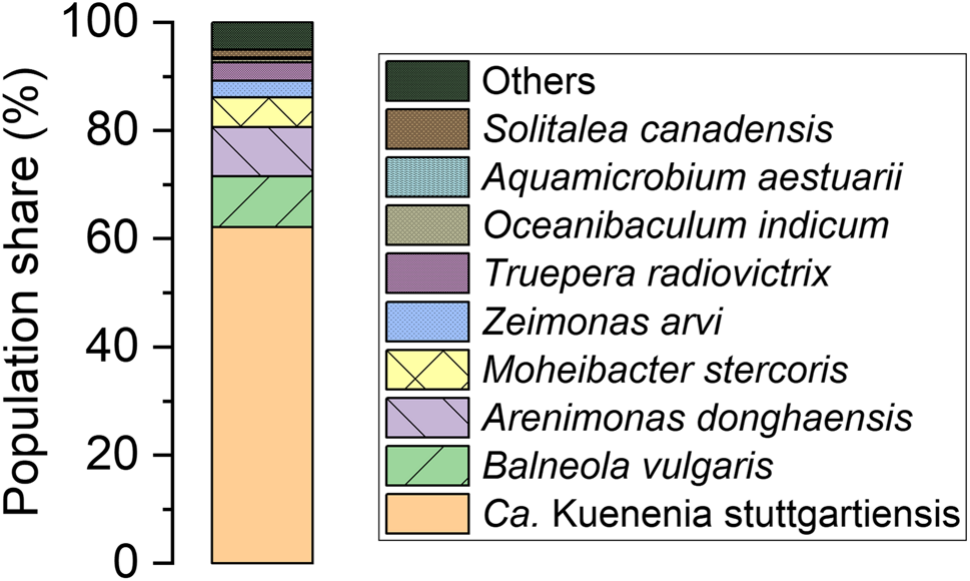
Community composition in the upscaled anoxic reactor as determined by 16S rRNA gene sequencing. *Ca.* K. stuttgartiensis strain CSTR1 (class *Ca*. Brocadiia), *Balneola vulgaris* (class *Balneolia*), *Arenimonas donghaensis* (class *Gammaproteobacteria*), *Moheibacter stercoris* (class *Falvobacteria*), *Zeimonas arvi* (class *Betaproteobacteria*), *Truepera radiovictrix* (class *Deinococci*), *Oceanibaculum indicum* (class *Alphaproteobacteria*), *Aquamicrobium aestuarii* (class *Alphaproteobacteria*), and *Solitalea canadensis* (class *Sphingobacteriia*)

As further support evidence, we determined the composition of the microbial populations in the 30-L reactor by 16S rRNA gene amplicon sequencing giving 78,922 quality-filtered sequences. These sequences were taxonomically classified and grouped into 80 operational taxonomic units. As many as 49,033 sequences, which relates to 62.1% of all determined sequences, were affiliated with *Ca.* Kuenenia strain CSTR1 (Fig. 3). Further abundant populations were *Balneola vulgaris* (9.4%, phylum *Balneolaeota*, class *Balneolia*), *Arenimonas donghaensis* (9.1%, phylum *Proteobacteria*, class *Gammaproteobacteria*), and *Moheibacter stercoris* (5.5%, phylum *Bacteriodota*, class *Flavobacteriia*).

### Anammox activity was inhibited by oxygen and recovered by sulfite

**Fig. 4:**
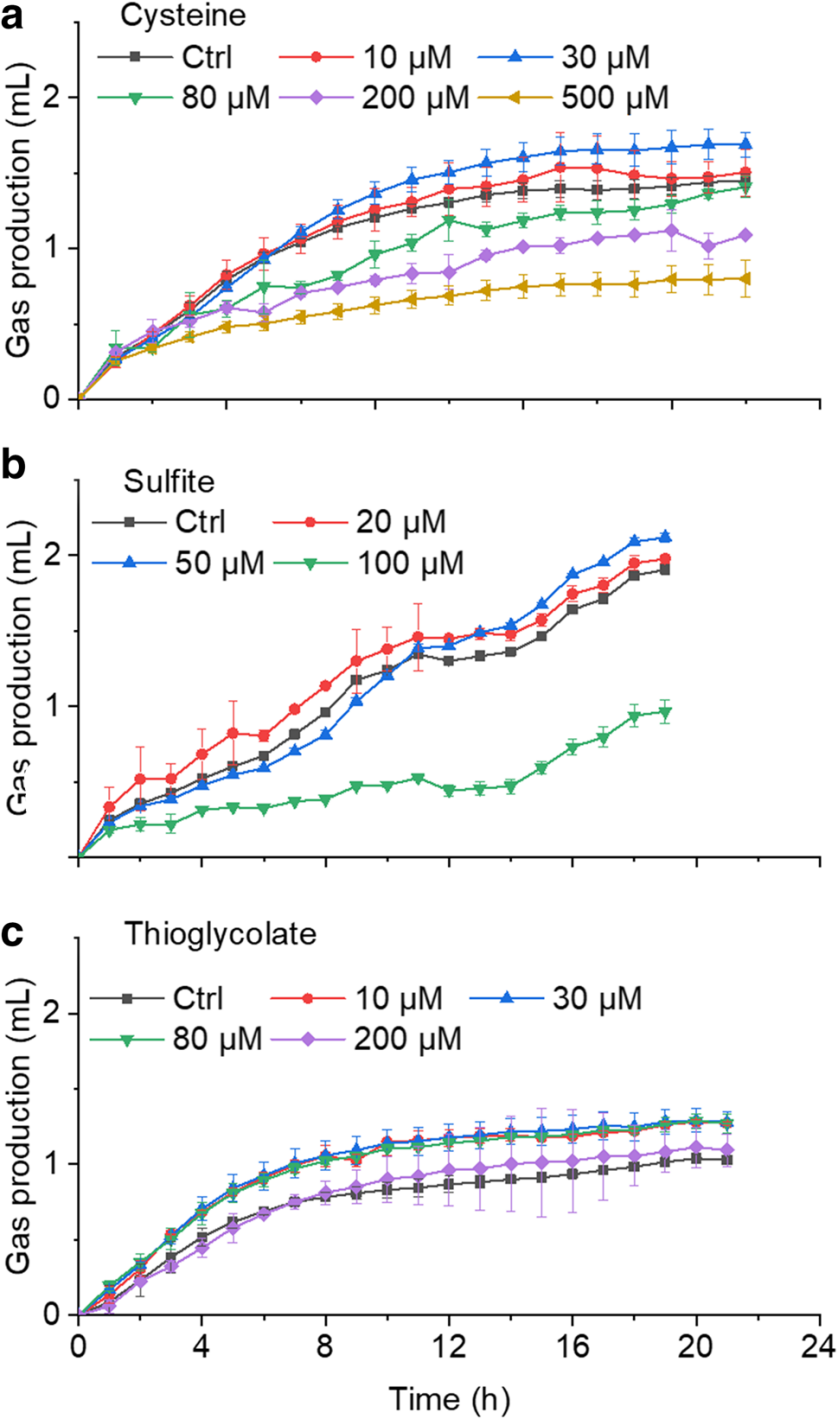
Tolerance of anammox activity towards three reducing agents. **a** Cysteine, **b** sulfite, and **c** thioglycolate at increasing concentrations. Activity of *Ca.* K. stuttgartiensis strain CSTR1 was monitored through gas pressure monitoring. Ctrl, control without sulfite/cysteine/thioglycolate. Data are presented as means ± SD (*n* = 3)

**Fig. 5:**
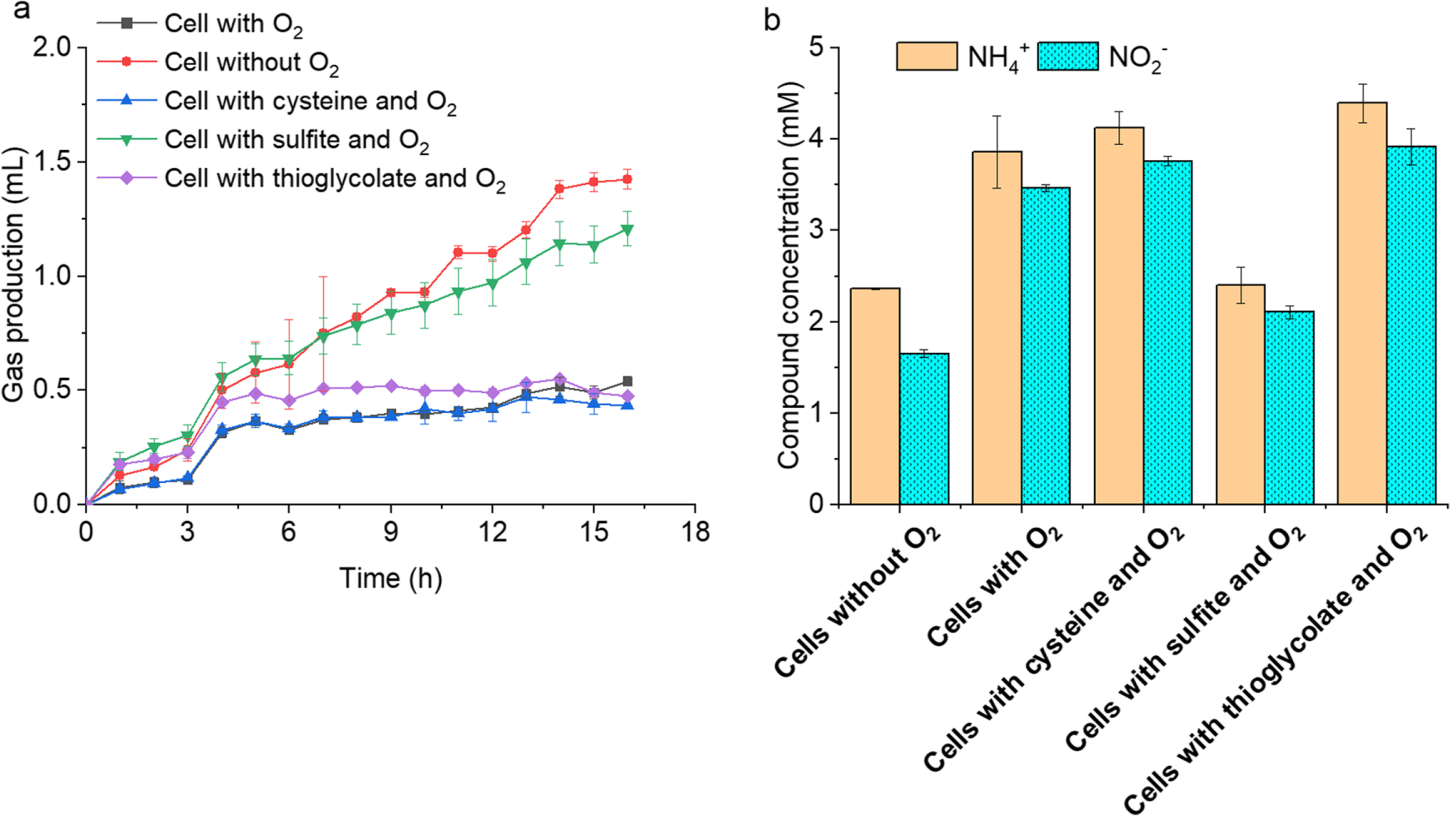
Inhibition of anammox activity of *Ca.* K. stuttgartiensis strain CSTR1 by oxygen and release of this inhibition by adding the reducing agents cysteine (30 µM), sulfite (30 µM), or thioglycolate (30 µM). **a** Gas production over 16 h of incubation; **b** remaining concentrations of ammonium and nitrite (mM) after 16 h of incubation. Data are presented as means ± SD (*n* = 3)

Anammox bacteria have been described to be strictly anoxic; however, it is not clear whether oxygen-inhibited activity can be rescued by reducing agents, such as cysteine, sulfite, or thioglycolate. In a preliminary experiment, we tested for possible direct inhibition of anammox activity by the reducing agents themselves. For that, anammox activity was quantified in whole-cell activity tests amended with the three reducing agents at different concentrations—but without oxygen (Fig. 4). Neither cysteine nor sulfite had any inhibitory effect on anammox activity at concentrations up to 30 and 50 µM, respectively. Inhibitory effects were observed for cysteine up from 80 µM and for sulfite from 100 µM. Thioglycolate up to 200 µM did not cause inhibition of anammox activity. We then tested whether the reducing agents at concentrations of 30 µM could rescue oxygen inhibition on anammox cells in a resting cell activity test (Fig. 5). Whereas sulfite recovered gas production (as proxy for N_2_ production), cysteine and thioglycolate did not (Fig. 5a). Ammonium consumption was reduced by 71%, 74%, and 85% in the negative control (with oxygen but without reducing agents), in cysteine, and in thioglycolate treatments, respectively (Fig. 5b). Incubation with 30 µM sulfite abated the oxygen-inhibitory effect, and almost no anammox activity was lost. In summary, these results reveal that oxygen is a potent but reversible inhibitor of anammox activity, and activity recovery is possible when an appropriate reducing agent such as sulfite is added to remove molecular oxygen. The added concentration of sulfite was low enough to envisage practicability even in industrial settings.

### Anammox activity is susceptible to antibiotics

**Fig. 6:**
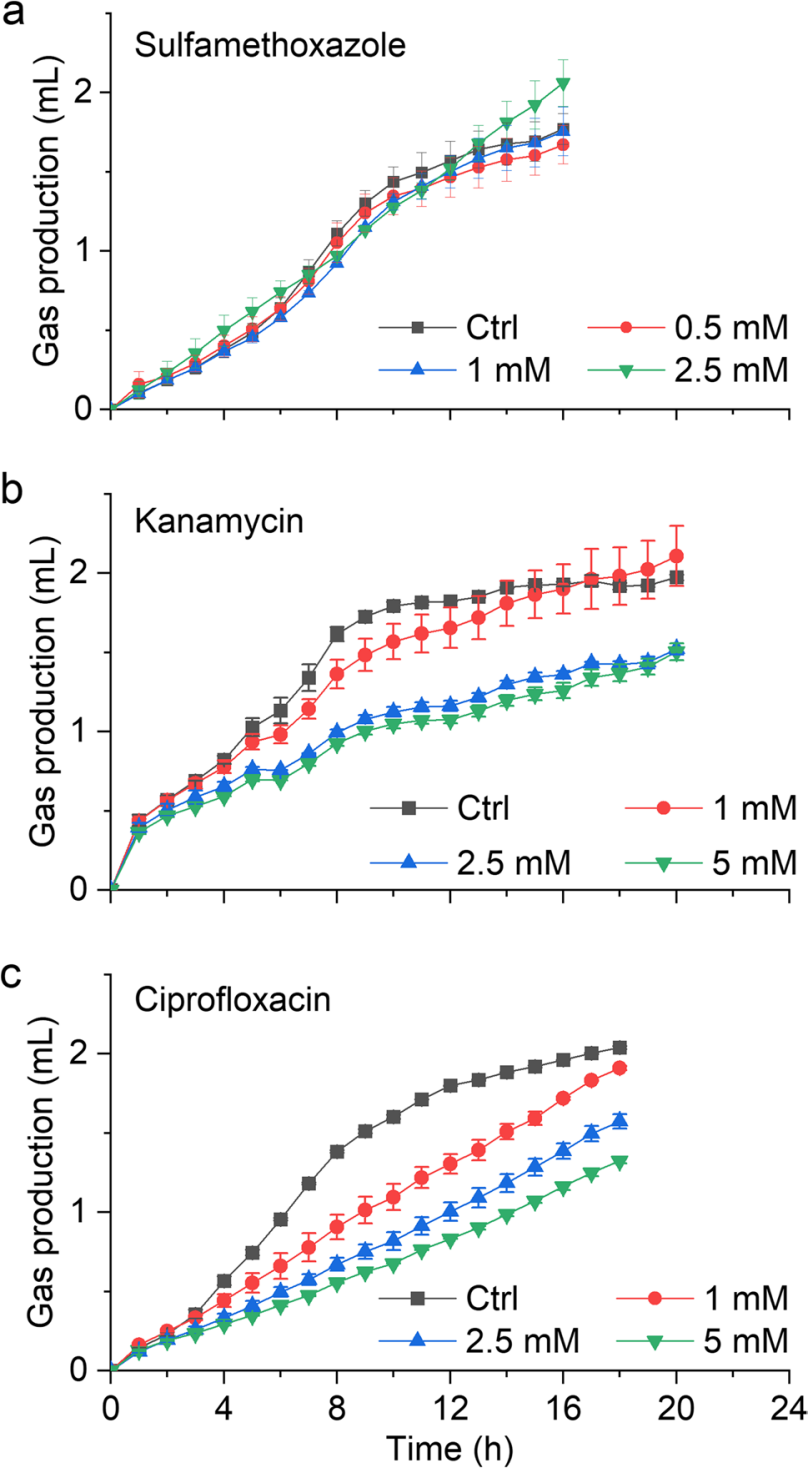
Effect of **a** sulfamethoxazole, **b** kanamycin, and **c** ciprofloxacin on anammox activity of *Ca.* K. stuttgartiensis strain CSTR1. Data are presented as means ± SD (*n* = 3)

The three antibiotics sulfamethoxazole, kanamycin, and ciprofloxacin had an inhibitory effect on anammox activity, when at relatively high concentrations in the millimolar range (Fig. 6), depending on antibiotics type and concentration. Sulfamethoxazole, at concentrations as high as 2.5 mM, did not impact activity. Kanamycin reduced anammox activity at concentrations above 1 mM, while ciprofloxacin reduced anammox activity at all of the tested concentrations; the inhibition increased with increasing concentrations of the antibiotics.

### Strain CSTR1 recovered anammox activity after 2 weeks of exposure to sulfamethoxazole and sulfite as determined in a growth test

The aforementioned data revealed that strain CSTR1 can tolerate certain levels of sulfamethoxazole and sulfite in short-term activity tests. The two compounds might be interesting additions for bacterial enrichment (to remove other bacteria) and for medium redox buffering, respectively. We therefore performed experiments to determine how these substances affect anammox activity in a long-term manner when the growth of the bacteria plays a role (Table S4). The inoculum was obtained from the 30-L reactor and transferred to sterile media bottles containing 20 µM sulfite or 1 mM sulfamethoxazole (5% inoculum; starting cell density 9.0 × 10^6^ cells mL^−1^). After 2 weeks, all nitrite was consumed in the positive control and in the cultures with amended sulfite. Conversely, in the cultures with sulfamethoxazole amendment, a small amount of nitrite remained after 2 weeks of incubation, but this nitrite was fully consumed by the end of the experiment. The ammonium concentrations decreased in a similar trend, while nitrate concentration increased steadily. The increase of nitrate concentration and cell density in the first week was low in all treatments. When we reduced tenfold the inoculum size (0.5%, starting cell density 9.0 × 10^5^ cells mL^−1^), the growth of strain CSTR1 was inhibited by both effectors (Table S4b).

### Fe_2_O_3_ and activated carbon enhanced anammox activity

**Fig. 7:**
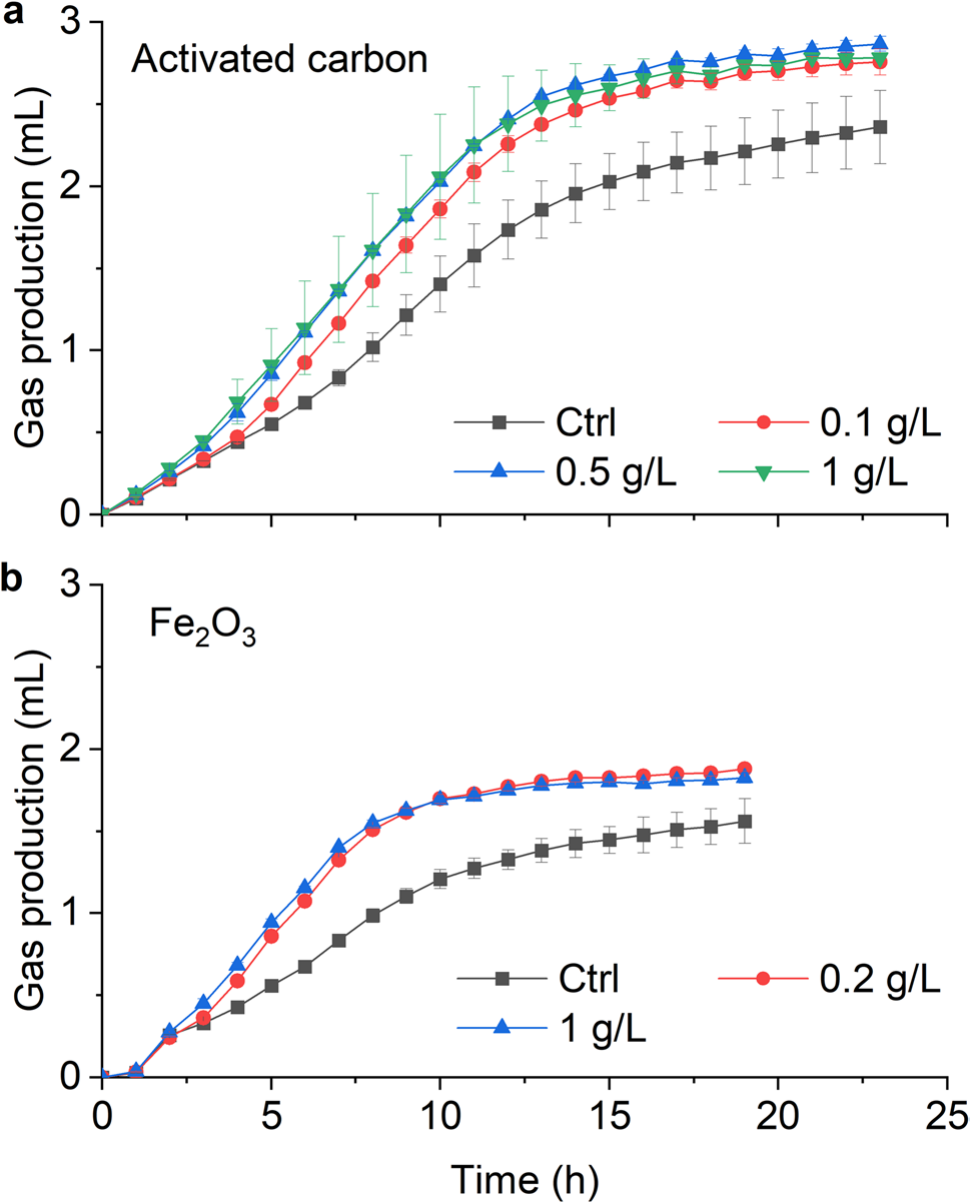
Stimulatory effect of **a** activated carbon and **b** Fe_2_O_3_ on the activity of *Ca.* K. stuttgartiensis strain CSTR1 determined by gas production. Data are presented as means ± SD (*n* = 3)

With the whole-cell activity test, we also evaluated compounds that could possibly stimulate anammox activity, i.e., activated carbon and ferric oxide (Fe_2_O_3_). Overnight incubation showed that the amendment with activated carbon or Fe_2_O_3_ enhanced anammox activity of strain CSTR1, as indicated by increased gas production (Fig. 7). After 20 h of incubation, gas production for the activated carbon amended bottles had reached 2.67 mL, and this slowly increased to 2.87 mL at the end of the experiment (Fig. 7a). Conversely, the control bottles without activated-carbon amendment had a gas production of 2.09 mL after 20 h, and this value increased to 2.36 mL at the end of the experiment. The initial rate of gas production in the activated carbon-amended treatment was 0.15 mL h^−1^, which was higher than that in the control bottles (0.12 mL h^−1^). No difference was detected between different amounts of activated carbon added, indicating that the lowest chosen concentration of 100 mg L^−1^ already achieved the maximum effect. Similarly, amendments of Fe_2_O_3_ increased the overall rate of gas production (Fig. 7b). The addition of Fe_2_O_3_ increased anammox activity from approximately 0.08 mL h^−1^ to 0.10 mL h^−1^ at 0.2 g L^−1^ Fe_2_O_3_ and 1 g L^−1^ Fe_2_O_3_, respectively. However, during the first four hours of incubation, almost no difference in gas production was verified between Fe_2_O_3_-amended cultures and the control cultures without Fe_2_O_3_; the difference was observable only after four hours of incubation. The increased activity with Fe_2_O_3_ was confirmed by enhanced ammonium consumption (Fig. S2). Manganese additions in both tested charge states, MnCl_2_ and MnO_2_, barely affected the activity of strain CSTR1 within the first 24 h of incubation (Fig. S3).

**Fig. 8:**
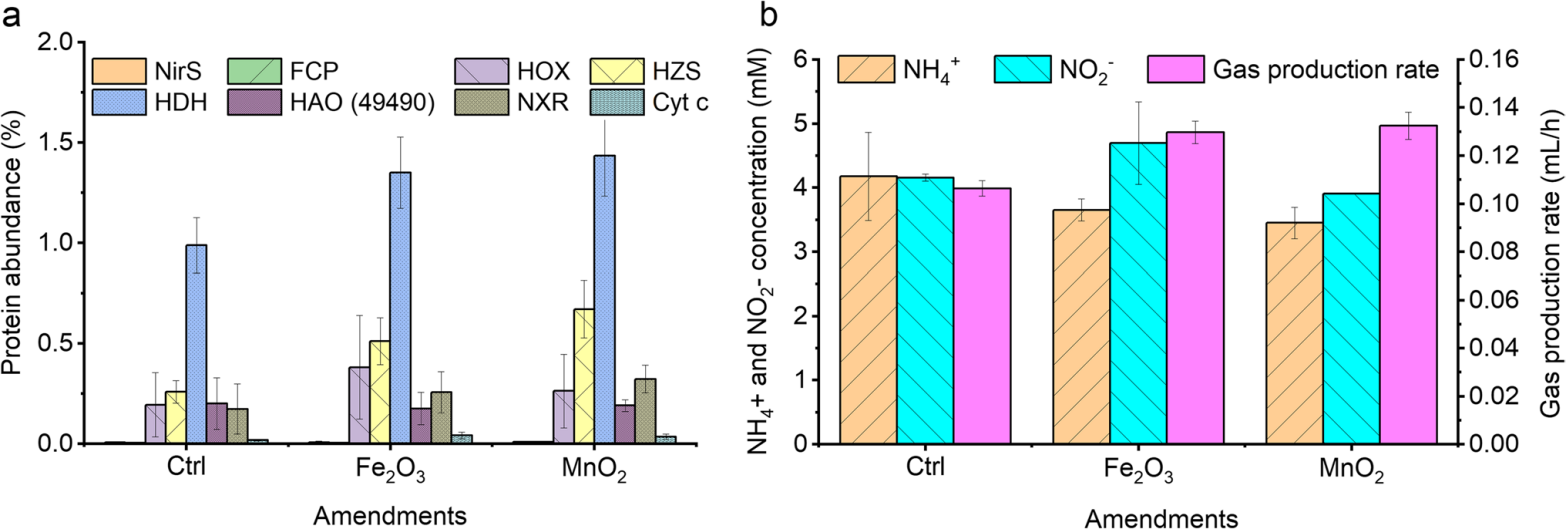
Estimation of the abundance of key anammox proteins in batches with or without Fe_2_O_3_ or MnO_2_ after 24 h of incubation. **a** Protein expression levels as an indicator of anammox metabolism. **b** Anammox activity determined by nitrogen production (pink bars) when the anammox culture was incubated with inorganic additions (0.2 g L^−1^ Fe_2_O_3_ or 0.1 g L.^−1^ MnO_2_). Ammonium and nitrite values show the concentrations remaining after incubation. FCP, putative nitrite reductase electron transfer iron-sulfur-cluster subunit (KsCSTR_46210); NirS, nitrite reductase (KsCSTR_33370); NXR, nitrite:nitrate oxidoreductase, (KsCSTR_08000, KsCSTR_07970, KsCSTR_07960); HOX, hydroxylamine oxidase (KsCSTR_43280); Cyt c, NapC/NirT family cytochrome c (KsCSTR_12840); HZS, hydrazine synthase (KsCSTR_28210, KsCSTR_28190, KsCSTR_12680); HDH, hydrazine dehydrogenase (KsCSTR_46980, KsCSTR_11820); HAO, putative hydroxylamine oxidoreductase HAO (KsCSTR_49490). Protein abundance represents the percentage of expressed proteins in the whole proteome, while the absolute abundances of anammox protein are shown in Table S5. Data are presented as means ± SD (*n* = 3)

Finally, we used a proteomics approach to analyze the expression levels of proteins reported to be involved in the respiratory anammox metabolism, in cells incubated with or without the potential inorganic electron acceptors Fe_2_O_3_ and MnO_2_. We hypothesized that changes in protein levels would be detectable after an incubation time of 24 h. The measured abundance of important enzymes involved in anammox reactions are summarized in Fig. 8a and Table S5. Several enzymes involved in the anammox process were differentially expressed with or without Fe_2_O_3_ or MnO_2_. The strong expression of anammox-related enzymes was consistent with the detected anammox activity, as evidenced by ammonium and nitrite consumption and gas production (Fig. 8b). The expression levels of anammox-related enzymes were significantly higher in cells incubated with Fe_2_O_3_ or MnO_2_ than in those in the control group incubated without these compounds. Consistent with previous studies, nitrite reductase (NirS), a candidate proposed to catalyze the conversion of nitrite to nitric oxide in the anammox reaction (Kartal et al. 2012), was expressed only at a low level. In contrast, nitrite:nitrate oxidoreductase (NXR) subunits were highly expressed and significantly (*p* ≤ 0.05) upregulated when incubated with Fe_2_O_3_ or MnO_2_. The putative hydroxylamine oxidoreductase HAO (KsCSTR_49490) and hydroxylamine oxidase (HOX) were expressed at a relatively high level. However, HAO was downregulated during incubation with Fe_2_O_3_ or MnO_2_, whereas HOX was significantly upregulated. Also, NapC/NirT family cytochrome c (KsCSTR_12840) was expressed at a relatively high level, and it was upregulated. Another enzyme predicted to be part of the electron transport chain (putative nitrite reductase electron transfer [4Fe-4S] cluster containing subunit, KsCSTR_46210) was expressed only at a low level. Hydrazine synthase (HZS) and hydrazine dehydrogenase (HDH) were the two most abundant proteins in the proteome, both of which were upregulated in response to Fe_2_O_3_ and MnO_2_.

## Discussion

### Reactor conditions and bacterial enrichment

Anammox bacteria are already used in wastewater technology to remove nitrogen from ammonium-rich side streams, but their notoriously slow growth and sensitivity to varying environmental conditions have hindered their broad application. Two recent studies have shown that the doubling time of *Ca.* K. stuttgartiensis can be reduced from approximately 3 weeks down to nearly 2 days, under appropriate conditions (Ding et al. 2018; Lotti et al. 2015), demonstrating that the intrinsic growth rate limitation of anammox bacteria is much weaker than anticipated. This directly leads to the main question of the current study, which environmental parameters limit the growth rate of anammox bacteria. We addressed this question by performing studies on a planktonic culture established in our laboratory. Growing in planktonic state is different from growing in wastewater treatment plants, where the cells grow in flocs within a rich microbial community. However, the investigation of planktonic cells allows to exclude indirect effects and to directly test anammox cells. This approach also excludes the influence of physicochemical parameter such as diffusion limitation.

Our study showed that the growth in planktonic form is not restricted to elaborated lab-scale glass reactors. Instead, we obtained stable operation for many months in a 35-L plastic container with 30 L of liquid culture, providing sufficient cell material for all subsequent experiments. No obvious limitations to further upscale planktonic cultivation were observed. We used the biomass in the effluent for biochemical experiments, but it is also feasible to produce commercial anammox inoculum via such a system. The direct use of such planktonic cultures in wastewater streams is currently not feasible owing to the low cell numbers and lack of biomass retention.

It has previously been reported that heme proteins, including the enzymes HDH, HZS, NirS, HAO, and cytochrome *bc*_*1*_, play crucial roles in the anammox process (Bi et al. 2014). Heme proteins make up approximately 30% of the total protein complement of *Ca.* K. stuttgartiensis, giving anammox bacteria their characteristic reddish color that gradually increases in intensity during enrichment (Kartal et al. 2012). We observed that, during planktonic growth, this red color can be confidently used as a proxy for the abundance and activity of anammox bacteria as proposed previously (Jafari Ozumchelouei et al. 2020; Meng et al. 2019). The photometric detection in the effluent allows reliable online monitoring of planktonic cultures.

Molecular community analysis demonstrated that *Ca.* K. stuttgartiensis maintained its dominant role in the community in the upscaled reactor. As suggested in previous studies, *Ca.* K. stuttgartiensis has geared its metabolism toward a low K_m_ for nitrite (Kartal et al. 2012), which indeed was continuously in the lower µM range in our reactor. The absolute number of anammox bacteria after 2 months of enrichment in the 30-L reactor was comparable to that obtained previously (Ding et al. 2018). Interestingly, lower bacterial diversity was found in the 30-L reactor compared to other reactors (van de Vossenberg et al. 2008). Low population shares of *Oceanibaculum indicum* (Lai et al. 2009; Liu et al. 2010) and *Aquamicrobium aestuarii* (Jin et al. 2013; Zhao et al. 2017), both of which are members of *Alphaproteobacteria*, were detected in the reactor and both were reported to reduce nitrate to nitrite. Their physiological role in the reactor could be the reduction of nitrate, which is produced as a side product by strain CSTR1 (Hosokawa et al. 2021).

### Reducing agents can support anammox activity

It is long known that oxygen inhibits anammox bacteria (Strous et al. 1997; Zhang and Okabe 2020). We here reproduced these results using planktonic cells that were not protected by facultative anaerobic bacteria acting as oxygen scavengers. In fact, oxygen inhibition was immediate and acted directly on the anammox process (not only on growth), possibly via oxidation of low-potential redox mediators in the cell. We also showed that inhibition of the anammox activity by oxygen was reversible and protectable by using reducing agents. In our experiment, 30 µM of sulfite was sufficient to protect the anammox metabolism from the inhibitory effect of oxygen in the headspace. The low rate of oxygen diffusion from the atmosphere to the liquid and the additional action of other bacteria in the community might have contributed to the result in which already very low concentrations of the reducing agent were present and had a protecting effect. Such a strong effect at low concentrations of sulfite is astonishing, but the implications for practical application could be strong because the amendment of low sulfite concentrations during the set-up phase of anammox plants are realistic also under industrial scale. It is worth noticing that we have come to this conclusion after experiments with a *Ca.* Kuenenia strain. We cannot generalize this finding to other anammox bacteria, such as *Ca.* Brocadia strains, that have often high population shares in operational anammox plants (Reino et al. 2018); however, the situation is similar—the anammox process is highly sensitive to oxygen and redox buffering should stabilize such systems. Commonly described amounts of sulfite used for cultivation were in the millimolar range and kept the redox potential below -110 mV even after exposure to air (Rothe and Thomm 2000). Conversely, cysteine hydrochloride and sodium thioglycolate were not able to recover the anammox activity. This was an unexpected result, since they are considered strong reducing agents with recognized reducing capacity (Rymovicz et al. 2011). Cysteine hydrochloride is widely used as a reducing agent owing to its low toxicity, and it is commonly used to prepare pre-reduced culture media for anoxic and strictly anaerobic bacteria (Fukushima et al. 2003). Sodium thioglycolate, however, reportedly needs heat activation to remove oxygen (Wagner et al. 2019). In addition, thioglycolate has been reported to be toxic (Mauerhofer et al. 2019); in a concentration of 0.01% (w/v, or 0.9 mM), it inhibited the growth of *Clostridium botulinum* (Smith and Pierson 1979), although 0.9 mM is much higher than the maximum concentration (200 µM) used in our work.

### Antibiotics in high concentrations inhibited anammox activity

The applied antibiotics sulfamethoxazole, kanamycin, and ciprofloxacin are described to act on folate biosynthesis, translation, and DNA replication (gyrase inhibitor), respectively (Gilbert et al. 2002). Therefore, all three antibiotics should primarily impact the growth of susceptible bacteria. However, we also applied the whole-cell activity test to determine whether the antibiotics would have inhibitory effects on the anammox enzyme apparatus. The inhibition of anammox activity was apparent only at concentrations in the millimolar range (1–5 mM). This result indicates that enzymatic anammox activity itself is not much impacted by these three antibiotics at concentrations occurring in real wastewater. Taken together the results from anammox growth and activity experiments, we conclude that concentrations of the investigated antibiotics in real wastewater are unlikely to reach a level that could be inhibitory to the anammox process.

### Activated carbon and Fe_2_O_3_ enhanced anammox activity

In our experiments, the addition of activated carbon significantly enhanced the anammox activity. We did not investigate the causes for this activity improvement, but it might have been due to enhanced enzymatic activity (Nguyen et al. 2014) or due to the immobilization of anammox bacteria (Tao et al. 2015). Similarly, degradation of the recalcitrant trace organic contaminants carbamazepine, diclofenac, sulfamethoxazole, and atrazine by laccase were improved by the addition of granular activated carbon (Nguyen et al. 2014).

We also revealed that Fe_2_O_3_ enhanced anammox activity. Fe(III) can serve as an electron acceptors (Strous et al. 2006), e.g., when coupled to anaerobic ammonium oxidation, a process described as “Feammox” (Desireddy and Pothanamkandathil Chacko 2021; Wan et al. 2022). In such a system, the reduction of Fe(III) to Fe(II) could take up the electrons from ammonium oxidation, whereas the oxidation of Fe(II) to Fe(III) could be supported by nitrate as an electron acceptor, effectively leading to an anammox-like process, in which nitrate is used as terminal electron acceptor to oxidize ammonium (Yang et al. 2020). We noticed an increased ammonium and reduced nitrite consumption in the presence of Fe_2_O_3_, suggesting that Fe_2_O_3_ might be used as an electron acceptor instead of nitrite. This further suggests that the addition of Fe(III) in the anammox system can improve ammonium-removal efficiency by enhancing growth of strain CSTR1 and providing additional energy. Furthermore, the addition of Fe(III) could promote heme biosynthesis (Huang et al. 2014) and, consequently, stimulate the anammox process via the facilitated biosynthesis of cytochromes and multi-heme enzymes (Ferousi et al. 2017; van Niftrik and Jetten 2012).

Manganese, like iron, was used by *Ca.* K. stuttgartiensis as a respiratory electron acceptor when formate was provided as the electron donor (Strous et al. 2006). Nevertheless, the results in our short-time whole-cell incubation experiments did not show a consistent and significant increase of the anammox activity in the presence of differently oxidized Mn species. Experiments running for prolonged periods of time, in which actual growth occurs, might render different results.

### Enzymes involved in the anammox process were differentially expressed with Fe_2_O_3_ or MnO_2_

Fe(III) and Mn(IV) are potential respiratory electron acceptors for *Ca.* Kuenenia strains (Strous et al. 2006) and have shown a strong effect on anammox activity in a reactor setup (Huang et al. 2014). Therefore, in an overnight whole-cell incubation assay, we investigated the differential protein expression after the addition of Fe_2_O_3_ or MnO_2_. Indeed, enzymes related to the anammox process were highly expressed in the presence of Fe_2_O_3_ or MnO_2_, with abundance levels correlating with the observed anammox activity. On the other hand, the putative nitrite reductase electron transfer protein ([4Fe-4S] cluster binding subunit) and NirS did not show increased abundance, indicating that they are not key factors in the metabolism with Fe_2_O_3_ or MnO_2_. Similar low abundance of these two proteins was found in previous studies when *Ca.* K. stuttgartiensis was stimulated with Fe(II) (Ding et al. 2021; Huang et al. 2014). NirS was hardly expressed at the transcriptional and protein levels compared to other key catabolic proteins in *Ca.* K. stuttgartiensis (Kartal and Keltjens 2016). Also, in another study from the same research group, these enzymes were barely detected (de Almeida et al. 2016), suggesting that other proteins catalyzed nitrite reduction.

In our study, several anammox-related protein complexes were strongly upregulated in the presence of Fe_2_O_3_ and MnO_2_. The NapC/NirT family cytochrome c (KsCSTR_12840) was expressed at a relatively high level in response to the effectors. Cytochrome c is hypothesized to bind to the HZS complex to shuttle electrons from an electron donor to hydrazine synthase (Smeulders et al. 2020). It was also hypothesized that, if this cytochrome c is involved in electron shuttling, it should be upregulated in response to the effectors (Akram et al. 2019). The upregulation of the NapC/NirT family cytochrome c and HZS in response to stimulators is an indication that there is an interplay between the expression of anammox-related proteins and the addition of inorganic substrates. Possibly, the respiration with Fe_2_O_3_ or MnO_2_ in the presence of the anammox substrates ammonium and nitrite causes ATP starvation, leading to the induction of anammox-related proteins. This would mean that strain CSTR1 was able to regenerate ATP via anaerobic respiration in parallel to the anammox reaction.

The two most expressed proteins (HDH and HZS) showed significantly higher abundance in the treated conditions than in the controls. HZS has a propeller-like architecture, which provides a structure for protein–protein interactions and binding of cofactors (Kartal and Keltjens 2016). HDH is an octaheme c-type cytochrome, which catalyzes the oxidation of hydrazine to dinitrogen gas and provides four electrons to replenish the electron pool (Bi et al. 2014; Kartal et al. 2012). Their high abundance suggests that the catalyzed reactions limit the overall pathway in vivo. The result in which addition of Fe_2_O_3_ or MnO_2_ led to even further expression suggests a physiological link between the anammox reactions in *Ca.* Kuenenia and the physiological use of other electron acceptors, such as Fe_2_O_3_ or MnO_2_.

In summary, the establishment of a 30-L upscaled reactor with strong planktonic anammox activity was successful, and further upscaling is realistic. Whole-cell activity tests with planktonic cells give fast and reliable data on environmental stress factors and activity-stabilizing compounds, but cultivation is still necessary for assessment of growth. Most importantly, we found that sulfite at concentrations as low as 30 µM can significantly support anammox bacteria by protecting from oxygen stress. We anticipate that the system can be used for further physiological characterizations and the design of resilient mixed microbial communities applicable in wastewater treatment.

## Supplementary Information

Below is the link to the electronic supplementary material.

Supplementary file1 (PDF 333 KB)

## Data Availability

Paired-end sequencing data of bacterial culture from the upscaled anammox reactor have been deposited into the NCBI Sequence Read Archive (SRA) and are publicly available under the BioProject accession number: PRJNA879996.
