# Supplementary material for: Effects of reducing, stabilizing, and antibiotic agents on “Candidatus Kuenenia stuttgartiensis”
Source: Appl Microbiol Biotechnol. 2023 Feb 8;107(5-6):1829–43. doi: 10.1007/s00253-023-12375-w (PMC10006275; doi:10.1007/s00253-023-12375-w)
Supplement: Supplementary file 1 — Supplementary file1 (PDF 333 KB) [file 253_2023_12375_MOESM1_ESM.pdf]

Supplementary Information

Effects of reducing, stabilizing, and antibiotic agents on "*Candidatus* Kuenenia stuttgartiensis"

Emea Ude[1], Jucelaine Haas[1,2], Kaysar Kayoum[1], Chang Ding[1*], Lorenz Adrian[1,3]

[1] Helmholtz Centre for Environmental Research – UFZ, Department Environmental Biotechnology, Permoserstraße 15, 04318 Leipzig, Germany

[2] Federal University of Technology - Parana - UTFPR Campus Dois Vizinhos, Biological Control Laboratory, Estrada para Boa Esperança km 4, 85660-000 Dois Vizinhos, Brazil

[3] Chair of Geobiotechnology, Technische Universität Berlin, Ackerstraße 76, 13355 Berlin, Germany

* corresponding author: chang.ding@ufz.de

Table S1. Recipe for the start-up medium for the 30-L reactor

In comparison with the feeding solutions used during the operation of the reactor (Table S2A and S2B), the start-up medium contained lower concentrations of nitrite and ammonium (3 mM, each). Correspondingly, 57 mM NaCl was added to the start-up medium to compensate for the low ionic strength. Sulfite was added to remove oxygen from the start-up medium, and therefore sulfate was omitted (sulfur source in the feeding medium).

Chemical	Final concentration	Amount
Deionized water		Top up to 28 L
NaCl	3.331 g/L	93.3 g
NH_4Cl	0.161 g/L	4.51 g
$NaNO_2$	0.207 g/L	5.80 g
$MgCl_2.6H_2O$	0.1 g/L	2.8 g
KCl	0.3 g/L	8.4 g
$CaCl_2$	0.011 g/L	0.317 g
KH_2PO_4	0.160 g/L	4.48 g
K_2HPO_4	0.405 g/L	11.34 g
$NaHCO_3$	0.84 g/L	23.5 g
Na_2SO_3	0.047 g/L	1.32 g
Se/W/Mo[a] (20,000 × stock)	2 ×	2.8 mL
Trace element[b] (20,000 × stock)	5 ×	7 mL
Vitamin[c] (1,000 × stock)	2 ×	56 mL

[a] The Se/W/Mo solution contained: Na_2SeO_3 (0.08 g L^{-1}), $Na_2WO_4 \cdot 2H_2O$ (0.16 g L^{-1}), $Na_2MoO_4 \cdot 2H_2O$ (0.72 g L^{-1}), and NaOH (0.5 g L^{-1}).

[b] The trace element solution contained: $FeCl_2 \cdot 4H_2O$ (30 g L^{-1}), $CoCl_2 \cdot 6H_2O$ (3.8 g L^{-1}), $MnCl_2 \cdot 4H_2O$ (2 g L^{-1}), $ZnCl_2$ (1.4 g L^{-1}), H_3BO_3 (0.12 g L^{-1}), $NiCl_2 \cdot 6H_2O$ (0.48 g L^{-1}), $CuCl_2 \cdot 2H_2O$ (0.4 g L^{-1}), in 0.1 M HCl. Sulfite was used to remove dissolved oxygen in the medium.

[c] The vitamin solution contained: biotin (20 mg L^{-1}), folic acid (20 mg L^{-1}), pyridoxine hydrochloride (200 mg L^{-1}), riboflavin (50 mg L^{-1}), thiamine hydrochloride (50 mg L^{-1}), nicotinic acid (50 mg L^{-1}), D-pantothenic acid hemicalcium salt (50 mg L^{-1}), p-aminobenzoic acid (50 mg L^{-1}), thioctic acid (50 mg L^{-1}), and cyanocobalamin (50 mg L^{-1}).

Table S2. Recipe of the two feeding solutions for the 30-L reactor

The two feeding solutions were mixed (1:1) into the 30-L reactor giving a final inflow concentration of 60 mM nitrite and 60 mM ammonium.

a. Recipe of the 120 mM nitrite feeding solution

Chemical	Amount
Deionized water	Top up to 50 L
Se/W/Mo[a]	10 mL
KH_2PO_4	16 g
K_2HPO_4	40.5 g
Na_2SO_4	8.52 g
$NaNO_2$	414 g
Vitamin solution[b]	100 mL
$NaHCO_3$	84 g

[a] The Se/W/Mo solution contained: Na_2SeO_3 (0.08 g L^{-1}), $Na_2WO_4 \cdot 2H_2O$ (0.16 g L^{-1}), $Na_2MoO_4 \cdot 2H_2O$ (0.72 g L^{-1}), and NaOH (0.5 g L^{-1}).

[b] The vitamin solution contained: biotin (20 mg L^{-1}), folic acid (20 mg L^{-1}), pyridoxine hydrochloride (200 mg L^{-1}), riboflavin (50 mg L^{-1}), thiamine hydrochloride (50 mg L^{-1}), nicotinic acid (50 mg L^{-1}), D-pantothenic acid hemicalcium salt (50 mg L^{-1}), p-aminobenzoic acid (50 mg L^{-1}), thioctic acid (50 mg L^{-1}), and cyanocobalamin (50 mg L^{-1}).

b. Recipe of the 120 mM ammonium feeding solution

Chemical	Amount
Deionized water	Top up to 50 L
Trace element[a]	25 mL
$MgCl_2 \cdot 6H_2O$	10 g
KCl	30 g
$CaCl_2 \cdot 2H_2O$	1.50 g
NH_4Cl	321 g
HCl (1 M)	100 mL

[a] The trace element solution contained: $FeCl_2 \cdot 4H_2O$ (30 g L^{-1}), $CoCl_2 \cdot 6H_2O$ (3.8 g L^{-1}), $MnCl_2 \cdot 4H_2O$ (2 g L^{-1}), $ZnCl_2$ (1.4 g L^{-1}), H_3BO_3 (0.12 g L^{-1}), $NiCl_2 \cdot 6H_2O$ (0.48 g L^{-1}), $CuCl_2 \cdot 2H_2O$ (0.4 g L^{-1}) in 0.1 M HCl.

Table S3. Whole-cell activity tests performed with resting cells: tested effectors, concentrations, and types of acquired data

Experiment	Effector	Description	Tested concentrations	Data acquired
Exp #1 and #2<br>Fig. 4 and 5	Oxygen (O_2)	Injected in gaseous form (air)	0.3 mL air per mL headspace volume	NH_4^+, NO_3^-, NO_2^- concentrations and N_2 production
	Cysteine<br>(reductant)	Added as solution dissolved in anoxic water	[µM] 0, 10, 30, 80, 200, 500	N_2 production
	Sodium sulfite<br>(reductant)	Added as solution dissolved in anoxic water	[µM] 0, 20, 50, 100	N_2 production
	Sodium thioglycolate<br>(reductant)	Added as solution dissolved in anoxic water	[µM] 0, 10, 30, 80, 200	N_2 production
Exp. #3<br>Fig. 6	Sulfamethoxazole<br>(antibiotic)	Added as powder	[mM] 0, 0.5, 1, 2.5	N_2 production
	Kanamycin<br>(antibiotic)	Added as powder	[mM] 0, 1, 2.5, 5	N_2 production
	Ciprofloxacin<br>(antibiotic)	Added as powder	[mM] 0, 1, ,2.5, 5	N_2 production
Exp #4 and #5<br>Fig. 7 and 8<br>Fig. S2	Activated carbon<br>(promoting activity)	Added as powder	[g L^{-1}] 0, 0.1, 0.5, 1	N_2 production
	Fe_2O_3<br>(promoting activity)	Added as powder	[g L^{-1}] 0, 0.2, 1	N_2 production and anammox-related protein expression
Exp #5<br>Fig. 8<br>Fig. S3	$MnCl_2$<br>(inhibiting activity)	Added as powder	[mg L^{-1}] 0, 100	NH_4^+, NO_3^-, NO_2^- concentrations
	MnO_2<br>(inhibiting activity)	Added as powder	[mg L^{-1}] 0, 100	NH_4^+, NO_3^-, NO_2^- concentrations and anammox-related protein expression

Table S4. Growth experiment to investigate the effect of sulfite and sulfamethoxazole (SMX) on the anammox activity and growth of strain CSTR1 **(a)** using an inoculation volume of 5%, leading to an initial cell density 9.0 × 10^6 cells mL^{-1}; **(b)** using an inoculation volume of 0.5% leading to an initial cell density 9.0 × 10^5 cells mL^{-1}. Activity was evaluated as decrease in nitrite concentration over time, and (non-stoichiometric) increase of nitrate concentration over time.

(a)

Parameter	Incubation time (days)	Negative control: bottles without cells	Positive control: standard CSTR1 cultures	Cultures with 20 µM sulfite	Cultures with 1 mM SMX
NO_2^- (mM)	7	4.94 ± 0.12	3.46 ± 0.11*	3.43 ± 0.21*	3.54 ± 0.29*
	14	5.10 ± 0.07	0.00 ± 0.00*	0.00 ± 0.00*	0.63 ± 0.80*
	21	4.87 ± 0.06	0.00 ± 0.00*	0.00 ± 0.00*	0.00 ± 0.00*
NH_4^+ (mM)	7	4.30 ± 0.17	3.26 ± 0.15*	3.05 ± 0.10*	3.14 ± 0.38*
	14	4.95 ± 0.09	2.46 ± 0.20*	2.60 ± 0.18*	2.57 ± 0.28*
	21	5.19 ± 0.56	2.65 ± 0.14*	2.80 ± 0.15*	2.28 ± 0.08*
NO_3^- (mM)	7	0.10 ± 0.00	0.40 ± 0.03^{ns}	0.35 ± 0.06^{ns}	0.36 ± 0.04^{ns}
	14	0.00 ± 0.00	0.80 ± 0.01*	0.79 ± 0.07*	0.71 ± 0.11*
	21	0.03 ± 0.00	1.02 ± 0.55*	0.73 ± 0.02*	0.95 ± 0.45*
Cell density (cells mL^{-1})	7	-	9.0 × 10^6	9.0 × 10^6	9.0 × 10^6
	14	-	1.4 × 10^{7ns}	1.2 × 10^{7ns}	1.1 × $10^{7\ ns}$
	21	-	2.2 × 10^7*	2.3 × 10^7*	1.3 × 10^7*

(b)

Parameter	Incubation time (days)	Negative control: bottles without cells	Positive control: standard CSTR1 cultures	Cultures with 20 µM sulfite	Cultures with 1 mM SMX
NO_2^- (mM)	14	5.93 ± 0.01	1.52 ± 0.14*	4.41 ± 0.16*	3.62 ± 0.08*
	21	5.90 ± 0.02	0.00 ± 0.00*	4.69 ± 0.23*	3.26 ± 0.10*
	25	6.03 ± 0.05	0.00 ± 0.00*	4.52 ± 0.58*	3.84 ± 0.18*
NH_4^+ (mM)	14	5.92 ± 0.18	2.40 ± 0.03*	5.35 ± 0.15^{ns}	5.41 ± 0.10^{ns}
	21	6.18 ± 0.02	1.31 ± 0.09*	5.27 ± 0.10*	5.03 ± 0.12*
	25	5.84 ± 0.15	1.44 ± 0.00*	4.42 ± 0.62*	4.46 ± 0.27*
NO_3^- (mM)	14	0.00 ± 0.00	0.53 ± 0.02*	0.08 ± 0.07^{ns}	0.19 ± 0.15^{ns}
	21	0.02 ± 0.01	0.72 ± 0.01*	0.07 ± 0.04^{ns}	0.30 ± 0.24*
	25	0.02 ± 0.01	0.65 ± 0.02*	0.09 ± 0.07^{ns}	0.30 ± 0.07*

Cell numbers in (**b**) were below the quantification threshold of our cell counting method, and therefore, cell numbers were not monitored for this experimental part. Data show means ± SD (n = 3). For statistical evaluation the values for nitrite, ammonium and nitrate in the cultures were compared with the values in negative control bottles for the respective time point. To identify significant increase of cell density in (**a**), the values after 14, 21, or 25 days were compared with the value after 7 days.

*Significant differences at $p < 0.05$ according to Tukey's test; ns, not significant.

Table S5. Estimation of the abundance of key anammox proteins in batches with or without Fe_2O_3 or MnO_2 after 24 h of incubation.

Proteins (Unit: intensity given by MS)	KsCSTR[a]	Treatments								
		Ctrl			MnO_2			Fe_2O_3		
HDH	46980	3.6×10^8	4.0×10^8	2.2×10^8	5.1×10^8	3.6×10^8	5.6×10^8	6.4×10^8	2.9×10^8	4.0×10^8
HzsA	28210	1.1×10^8	5.3×10^7	1.2×10^7	2.4×10^8	1.5×10^8	3.0×10^8	2.6×10^8	7.4×10^7	1.6×10^8
HzsB	28190	1.3×10^8	1.0×10^8	2.3×10^7	2.1×10^8	1.5×10^8	3.1×10^8	2.7×10^8	9.0×10^7	1.9×10^8
HzsC	12680	1.7×10^8	9.1×10^7	1.4×10^7	1.5×10^8	1.0×10^8	2.1×10^8	1.8×10^8	5.9×10^7	1.1×10^8
NarG	08000	1.9×10^8	1.0×10^8	3.3×10^7	2.2×10^8	1.5×10^8	1.9×10^8	2.0×10^8	1.1×10^8	1.2×10^8
NarI	07960	3.3×10^7	1.3×10^7	6.0×10^6	3.4×10^7	2.5×10^7	4.5×10^7	4.6×10^7	1.8×10^7	2.6×10^7
NarH	07970	5.3×10^7	3.2×10^7	9.4×10^6	8.6×10^7	5.3×10^7	8.0×10^7	9.0×10^7	4.7×10^7	4.7×10^7
HOX	43280	1.1×10^8	2.8×10^7	3.5×10^7	6.7×10^7	3.1×10^7	1.4×10^8	2.0×10^8	5.0×10^7	9.3×10^7
HAO	49490	9.9×10^7	6.2×10^7	2.1×10^7	5.6×10^7	5.0×10^7	6.6×10^7	6.7×10^7	2.5×10^7	6.7×10^7
NirS	33370	3.2×10^6	2.3×10^6	5.3×10^5	2.2×10^6	n.q.	3.5×10^6	4.3×10^6	3.9×10^5	1.2×10^6
FCP	46210	1.1×10^6	n.q.	n.q.	2.4×10^6	1.7×10^6	4.5×10^6	2.1×10^6	1.2×10^6	1.8×10^6
Cytochrome c	12840	7.3×10^6	5.7×10^6	4.7×10^6	9.7×10^6	7.7×10^6	1.5×10^7	1.8×10^7	8.6×10^6	1.1×10^7
Total protein detected		4.2×10^9	2.5×10^9	1.0×10^9	4.2×10^9	2.9×10^9	5.2×10^9	5.1×10^9	2.0×10^9	3.1×10^9

Note: the experiments were performed in triplicates. FCP: putative nitrite reductase electron transfer iron-sulfur -cluster subunit, NirS: nitrite reductase, NXR: nitrite oxidoreductase, HOX: hydroxylamine oxidase, HZS: hydrazine synthase, HDH: hydrazine dehydrogenase, HAO: the putative hydroxylamine oxidoreductase HAO (KsCSTR_49490). n.q.: not quantifiable

[a] The column "KsCSTR" gives the locus tag in the genome (NCBI genome accession number CP049055.1), e.g. KsCSTR_49490.

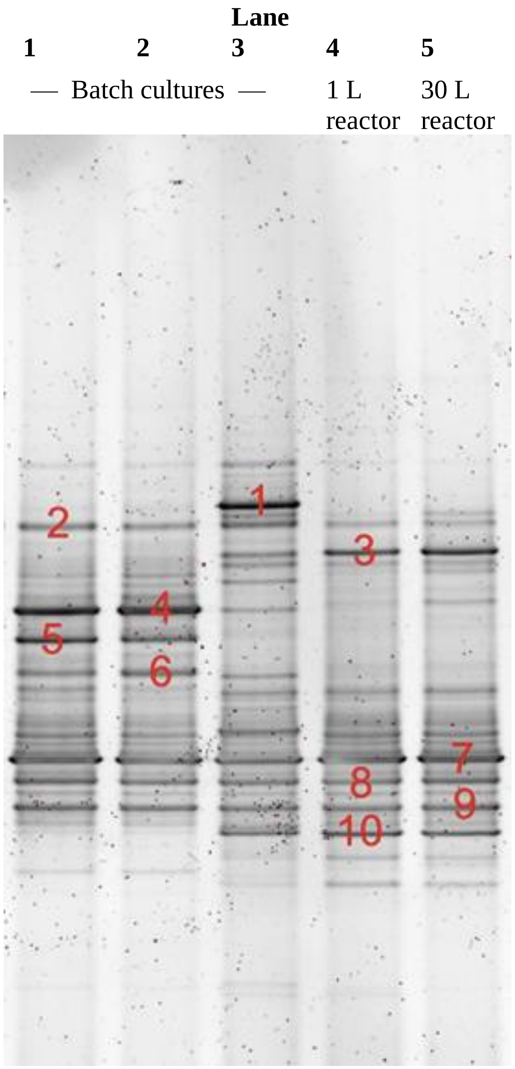

Band	Closest match	Identity (%)	Organism[a]
1	NR_025510.1	98	*Alicycliphilus denitrificans*
2	*(Bad sequencing result)*		
3	*(Bad sequencing result)*		
4	NR_165713.1	98	*Mesorhizobium denitrificans*
5	*(Bad sequencing result)*		
6	*(Bad sequencing result)*		
7	CP049055.1	99	*Ca.* K. stuttgartiensis strain CSTR1
8	CP049055.1	99	*Ca.* K. stuttgartiensis strain CSTR1
9	CP049055.1	99	*Ca.* K. stuttgartiensis strain CSTR1
10	*(Bad sequencing result)*		

[a] determined by BLASTN against the NCBI nt database

Fig. S1 DGGE analysis of mixed anammox cultures containing strain CSTR1. Lanes 1–3: DNA extracted from three parallel batch cultures (DNA extraction from 1-mL volume); lane 4: DNA extracted from the 1-L laboratory reactor that was used as inoculum for the 30-L reactor (DNA was extracted from 0.2 mL); lane 5: DNA extracted from 0.2 mL of the culture in the up-scaled 30-L reactor. Band 7 was the strongest band as directly seen under UV light. Owing to overexposure, there was a whitish area inside the band. The multiple bands for *Ca*. K. stuttgartiensis (lane 7–9) might be due to the use of degenerate base pairs in both the forward and the reverse PCR primers for DGGE. The genome of *Ca*. K. stuttgartiensis encodes only one copy of the 16S rRNA gene.

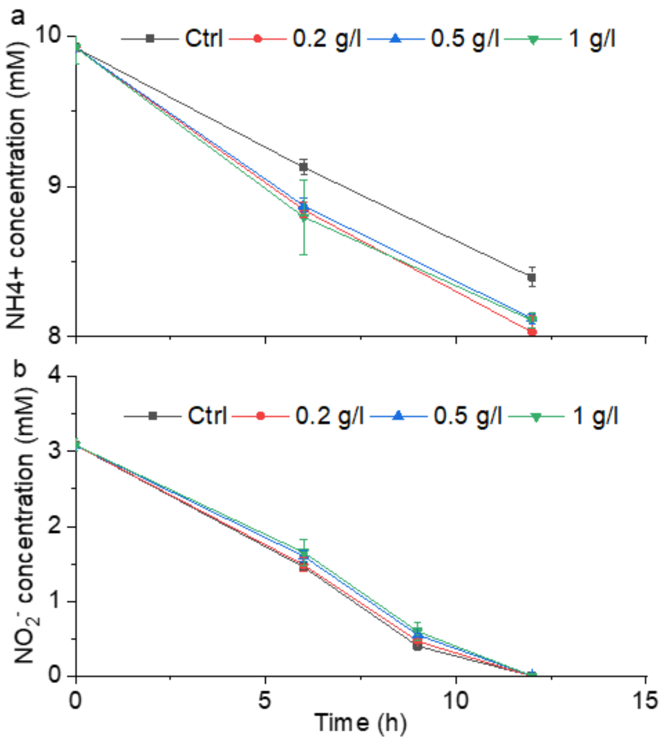

Fig. S2 Effects of Fe_2O_3 additions (0.2-1 g L^{-1}) on the remaining concentrations of (**a**) NH_4^+ and (**b**) NO_2^- during incubation with strain CSTR1. Initial concentrations were 10 mM NH_4^+ and 3 mM NO_2^-. Ctrl, positive control without Fe_2O_3. Shown are means of triplicates ± SD.

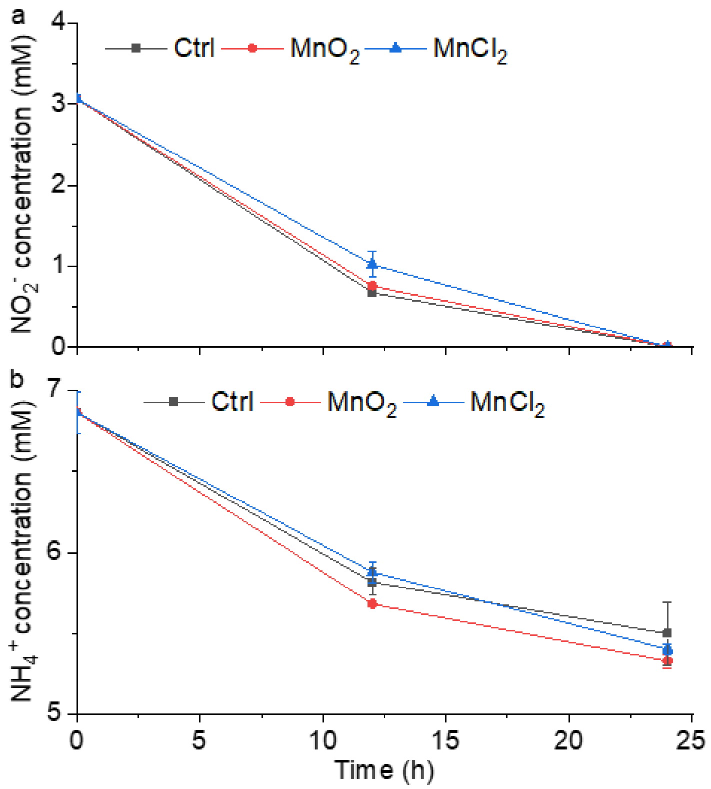

Fig. S3 Effects of manganese ions of oxidation state +IV (MnO_2) and oxidation state +II ($MnC_2{\cdot}4H_2O$) on (**a**) NO_2^- and (**b**) NH_4^+ concentrations after overnight incubation with strain CSTR1. Shown are the remaining concentrations of NH_4^+ and NO_2^- (mM). Initial concentrations were 3 mM NO_2^- and 7 mM NH_4^+. Ctrl, positive control without manganese. $MnC_2{\cdot}4H_2O$ was added at a concentration of 0.5 mM. MnO_2 was added at a concentration of 100 mg L^{-1}.
